# Elucidating the role of intrinsic adenosine A1 receptors in acute alcoholism using human-induced pluripotent stem cell-derived hepatocytes

**DOI:** 10.1042/BSR20231682

**Published:** 2024-03-21

**Authors:** Takako Nagata, Yuning George Huang

**Affiliations:** 1 NT Biologics, Gaithersburg, MD, U.S.A.; 2 National Institute of Diabetes and Digestive and Kidney Diseases, National Institutes of Health, Bethesda, MD, U.S.A.

**Keywords:** acute alcoholism, adenosine A1 receptors, apoptosis, hepatitis, metabolism

## Abstract

Acute alcoholic hepatitis (AAH) from binge drinking is a serious disease. It is associated with a high mortality rate, especially among young adults. Apoptosis is known to be a primary cause of liver damage, and it can be induced by either intrinsic signaling pathways or by reactive oxygen species (ROS). Adenosine A1 receptors (ADORA1) are known to be involved in ethanol metabolism; however, underlying mechanism is not well understood. For investigating how the intrinsic ADORA1 function in ethanol metabolism in normal human hepatocytes without interference by extrinsic molecules, primary hepatocytes pose a challenge, due to unavoidable contamination by other kinds of cells in the liver. Also, they are difficult to culture stably. As a novel alternative, hepatocytes derived from human-induced pluripotent stem cells were employed because they display similar function to primary hepatocytes and they can be stably cultured. The dynamics and integrity of signal transduction mechanisms were investigated by following chronological changes in gene expression. This shed light on how and when the ADORA1 function and on causal relationships between the pathways and clinical symptoms. The findings of the present study shows that ADORA1 are most activated soon after exposure to ethanol, and transfection of small interfering RNA targeting ADORA1-messenger-RNA (ADORA1-siRNA) into the hepatocytes significantly suppresses production of actin protein and ROS. It suggests that ADORA1 in the liver contribute to apoptosis in acute alcoholism through both intrinsic pathway and ROS activity. Also, actin that is abundant in the cells could be an appropriate biomarker evaluating hepatic function status.

## Introduction

Acute alcoholism from binge drinking has been a persistent problem worldwide. It especially targets youth, and the significantly high mortality rate destroy families while sacrificing lifetimes of human potential. So far, radical treatments have not been established and palliative therapies are sadly the rule. Moreover, alcohol induced acute hepatitis is often followed by fulminant hepatitis, where 1-month mortality rates are 40–50% [1]. According to the Centers for Disease Control and Prevention in the United States, during 2015–2019, excessive alcohol use was responsible on average for more than 140,000 deaths annually. More than 40% were tied to binge drinking.

Adenosine receptors are widely distributed and play vital roles in our bodies. Their expression is highly varied in each organ. Expression of adenosine A1 receptors (ADORA1), is highest in neurons; while the liver displays far lower expression – one of the lowest among tissues. Still, it was reported that ADORA1 plays a role in increasing ethanol-mediated hepatic steatosis by activating hepatic stellate cells [2].

Reactive oxygen species (ROS) are known to increase under oxidative stress and to cause apoptosis independently from caspase 3 involved pathways. Hence, accumulation of ROS in response to inflammation should also facilitate apoptosis. Induced apoptosis results in damage to nucleic acids, proteins, and membrane lipids in pathological conditions such as alcoholic hepatitis.

Hepatocytes exposed to sudden toxic concentrations of ethanol are subject to apoptosis via both intrinsic (mitochondria-mediated) and extrinsic (receptor-mediated) pathways. Hence, we investigated how ADORA1 contributes to ethanol metabolism, using HepG2 human hepatoma cell lines and ADORA1 knockout mice. Treatment of hepatocytes with ethanol in rats induces activation of c-Jun N-terminal kinase [3] that, in turn, activates some caspases which are pro-apoptotic enzymes. Caspase 3 is known to be typical among them and its activation level clearly reflects the severity of apoptosis.

The results (Supplementary Figure S1) were officially presented as poster 294 at the 35th Annual Scientific Meeting of the Research Society on Alcoholism in 2012 [4]. In summary, ADORA1 apparently plays a robust role in the up-regulation of ethanol metabolism in hepatocytes both in intrinsic and extrinsic pathways despite its low expression in the liver. As caspase3 is activated in both pathways, in order to figure out to which pathway(s) ADORA1 contributes in normal human hepatocytes following ethanol surge, primary hepatocytes were considered. However, it is technically challenging to isolate hepatocytes from other types of cells in liver, and to stably culture them. Hepatocytes derived from human-induced pluripotent stem cells (iPSC) were employed in this study to exclude the possibility of contamination, and also because they are stable in culture.

The RNA interference (RNAi) system down-regulates specific gene expressions by double-stranded RNA [5]. Following this discovery, it was reported that synthetic small interfering RNA (siRNA) could induce RNA-interference in mammalian cells [6].

In the present study, the kinetics of gene expression both in RNA and protein was investigated following exposure to a high concentration of ethanol in order to elucidate mechanisms where ADORA1, acts as a key molecule ethanol-induced metabolism associated with apoptosis, and in order to identify symptoms of acute alcoholic hepatitis that individual molecules and their involved pathways contribute to. Simultaneously, small interfering RNA targeting ADORA1-messenger-RNA (ADORA1-siRNA) was examined to see whether it suppresses the effects of ADORA1 in ethanol-induced cascades. ROS assay was also performed in order to verify this suppressive effect of ADORA1-siRNA.

## Materials and methods

### Reagents

Small interfering RNAs (siRNAs) to ADORA1, GAPDH, and Negative Control were purchased from Ambion (Austin, TX, U.S.A.). Lipofectamin (Lipofectamine RNAiMAX) was purchased from Invitrogen (Carlsbad, CA, U.S.A.). Tween 20 and bovine serum albumin-fraction V (BSA) were purchased from RPI (Mount Prospect, IL, U.S.A.). Rabbit anti-ADORA1 antibody was purchased from Sigma-Aldrich (Saint Louis, MO, U.S.A.). Mouse anti-actin antibody was purchased from Proteintech (Rosemont, IL, U.S.A.). Mouse anti-caspase 3 and anti-GAPDH antibodies were purchased from Santa Cruz Biotechnology (Dallas, TX, U.S.A.). Goat anti-rabbit Poly-HRP and goat anti-mouse IgG (H+L) Poly-HRP secondary antibodies were purchased from Invitrogen. Pre-casted 4–20% gradient gels, polyvinylidene fluoride (PVDF) membrane and other laboratory reagents were purchased from Bio-Rad (Hercules, CA, U.S.A.).

### Cell culture

Human hepatocytes derived from the Cellartis human induced pluripotent stem cell line 18 (ChiPSC18) were seeded and cultured at 1 × 10^5^ /well and 150 μl/well in 96-well plates with hiPS-HEP Medium (the Cellartis® Enhanced hiPS-HEP v2 Kit) according to its user manual (Takara Bio Europe AB, Sweden, catalog # Y10134). This cell line was derived from skin fibroblasts from a healthy volunteer (81 kg/175 cm), a 32-year-old adult male human (European/North African). HLA typification data: HLA-A*23:01; HLA-B*07:02, HLA-B*49:01; HLA-C*07:01, HLA-C*07:02; HLA-DRB1*04:06, HLA-DRB1*07:01; HLA-DQB1*02:02, HLA-DQB1*04:02; HLA-DPB1*03:01, HLA-DPB1*04:01.

### ADORA1-siRNA transfection into hepatocytes

During the second week of culture, the cells were cultured with or without ethanol 100 mM. ADORA1-siRNA was independently transfected into the hepatocytes with Lipofectamine RNAiMAX Reagent (Invitrogen) according to manufacturer’s protocol (Silencer Select siRNAs).

### Total RNA isolation and quantitative PCR

The hepatocytes cultured for 1.5, 3 or 6 h after the addition of ethanol were readily dissolved in TRIzol (Invitrogen) after the harvest and total RNA was purified according to the manufacturer’s instructions using RNA Clean & Concentrator-5 (Zymo Research, Irvine, CA, U.S.A.). The total RNA was quantified by Qubit 4 Fluorometer (Thermo Fisher Scientific, Waltham, MA, U.S.A.) and RNA integrity was assessed by 4150 TapeStation instrument G2992A (Agilent, Santa Clara, CA, U.S.A.). cDNA was generated on My Cycler (Bio-Rad) using Oligo(dT)20 Primer (Invitrogen) following manufacturer’s instructions. The cDNA was subjected to quantitative real-time PCR with PowerUp SYBR Green Master Mix (Applied Biosystems, Thermo Fisher Scientific) on 7500 Real-Time PCR System (Applied Biosystems). The sequences of the PCR oligonucleotide primers are listed in Table 2. The quantitative PCR experiments were repeated at least three times.

**Table 1:** Catalogue of major genes/proteins expressed in response to ethanol stimulation

Gene name	Function	Regulation	Assumed outcome
CDC42BPG^1^	Actin related	Up-regulation	Actin formation
ARHGEF26^1^	Actin related	Up-regulation	Actin formation
ID1^3^*	Actin related	Up-regulation	Actin assembly
OXT^2^	Inflammation related	Up-regulation	Up-regulating prostaglandin secretion
PTGDR2^2^	Inflammation related	Up-regulation	Facilitating inflammation responses
KNG1^2^*	Inflammation related	Up-regulation	Release of prostaglandins
SERPINE1^2^*	Inflammation related	Down-regulation	Stimulating inflammation
CEBPB^2^*	Inflammation related	Down-regulation	Aggression of acute-phase reaction
ID1^3^*	Apoptosis	Up-regulation	Up-regulating apoptosis
DDIT4^3^	Inhibition of the activity of the mammalian target of rapamycin complex 1 (mTORC1).	Up-regulation	p53/TP53-mediated apoptosis
IER3^3^	ERK1/2 activation	Down-regulation	Facilitating apoptosis
CTGF^3^	Activation of ERK1/2 and JNK	Down-regulation	Facilitating apoptosis
IGFBP1^3^*	Activation of AKT signaling pathway	Down-regulation	Facilitating apoptosis
HABP2^7^	Coagulation factor VII- activating	Up-regulation	Forming blood clots
KNG1^7^*	Blood coagulation	Up-regulation	Forming blood clots
SERPINE1^7^*	Thrombin inhibitor	Down-regulation	Forming blood clots
SLC2A1^4^	Glucose uptake	Down-regulation	Reducing glucose uptake
LYZ^4^	Glycogen release from lysosomes, becoming a final energy source	Down-regulation	No purge of glycogen, worsening Starvation state
IGFBP1^4^*	Glucose uptake	Down-regulation	Suppressing glucose uptake
CEBPB^4^*	Gluconeogenesis	Down-regulation	Down-regulation of gluconeogenesis
PHLDA2^4^	Controlling glycogen storage	Down-regulation	Down-regulation of glycogenolysis
SOAT2^5^	Secretion of cholesteryl esters	Down-regulation	Retaining of lipids in the cells
GK^5^	Controlling glycerol uptake	Down-regulation	Facilitating steatosis
NOXA1^6^	NADPH oxidase producing superoxide	Up-regulation	Inducing ROS production

*: Molecules having more than one function have repeat entries for each function. Superscript codes for each functional group are as follows: 1: actin formation related; 2: inflammation related; 3: apoptosis related; 4: Energy related; 5: Steatosis related; 6: ROS related; 7: blood coagulation related.

**Table 2:** The sequences of primer pairs

Gene	Forward (5′-3′)	Length (bp)	Reverse (5′-3′)	Length (bp)
ADORA1	CCTCCATCTCAGCTTTCCAG	20	AGTAGGTCTGTGGCCCAATG	20
Caspase 3 full length	GGCACAAAGCGACTGGAT	18	TGGCACAAAGCGACTGGAT	19
Casp-3p17	TGGAATTGATGCGTGATGTT	20	GGCAGGCCTGAATAATGAAA	20
GAPDH	CCCTGGCCAAGGTCATCC	18	TGATGGCATGGACTGTGGTC	20

### Next-Generation Sequencing (NGS) for gene expressions

Total RNAs obtained above were sent to Novogene (Beijing, China) which prepared RNA library and transcriptome sequencing using Illumina NovaSeq 6000. Differential gene expression was then calculated with DESeq2. Genes with adjusted *P*-value < 0.05 and |log2(FoldChange)| > 0 were considered to be differentially expressed. The reference genome was Homo Sapiens (GRCh38/hg38). The data obtained were analyzed using NovoSmart (Novogene) developed based on R shiny, Excel, Cytocscape, UniProt, and KEGG PATHWAY Database.

### Western blotting

The hepatocytes cultured for 2 days after the addition of ethanol were harvested and their cell pellets underwent the procedures as previously described in detail [7]. Protein concentrations were measured by Qubit 4 Fluorometer (Thermo Fisher Scientific) and by NI™ (Non-Interfering™) Protein Assay Kit (G-Biosciences, St Louis, Missouri, U.S.A.). Protein samples reduced with β-mercaptoethanol at 3% (Sigma-Aldrich) (approximately 30 μg for ADORA1 and actin, 20 μg for caspase 3) were loaded per lane on 4–20% gradient gels (Bio-Rad) along with rainbow markers (Amersham, Buckinghamshire, U.K.). Gels were run cold at constant 90 volts for an hour and a half. The separated proteins were transferred cold onto polyvinylidene fluoride (PVDF) membrane (Bio-Rad) at constant 300–350 mA for approximately 1.5 h. The membranes were blocked for 1 h in blocking buffer (3% BSA-PBS) on an orbital shaker (Benchmark, Tempe, AZ, U.S.A.) at ambient temperature. Following this, the membranes were incubated in PBS with Tween 20 at 0.1% (PBST) containing each one of the primary Abs (anti-ADORA1 diluted to 1:1000, anti-GAPDH diluted to 1:100, anti-actin diluted to 1:16,000, and anti-caspase 3 diluted to 1:200) at 6 degrees overnight. Following this, each membrane was washed four times for 4 min each in a washing buffer (0.1% Tween 20 in PBS). To enhance signals, poly-HRP conjugated secondary antibodies were employed (Mishra et al., 2019). Next, the membrane was incubated for 1 h with each one of the secondary antibodies (goat anti-rabbit PolyHRP and goat anti-mouse IgG (H+L) Poly-HRP) at 1:10000 dilution in 1% fat-free-milk PBST on the orbital shaker at ambient temperature. The incubated membranes were washed four times for 4 min each in washing buffer (Tween 20 at 0.1% in PBS), and developed with ECL Prime (Amersham). The fluorescence of the membrane bands was quantified by measuring their total fluorescence signals and analyzed using Amersham Imager 680 and ImageQuant TL (GE Healthcare, Chicago, IL, U.S.A.). The Western blot experiments were repeated at least three times.

### ROS assay

The hepatocytes were cultured at 1 × 10^5^ /well and 150 μl/well in black, clear-bottom, tissue culture-treated 96-well plates (Corning, Corning, NY, U.S.A.). First, the plates were added 50 μl/well using cold Hepatocyte Coating in Cellartis Enhanced hiPS-HEP Thawing and Plating Kit (Takara Bio Europe AB, catalog # Y10132), then incubated at room temperature for 60 min. Following removal of the Hepatocyte Coating from the wells just before seeding, the cultured cells were subjected to dihydroethidium (hydroethidine or DHE) based ROS assay, according to manufacturer’s protocol (Cayman Chemical, Ann Arbor, MI, U.S.A.). The hepatocytes were cultured for 3 or 48 h at 100 mM of ethanol with or without the addition of ADORA1-siRNA. Antimycin A was used as the positive control and N-acetyl cysteine as the negative control. The fluorescence was measured using an exciting wavelength of 485 nm and an emission wavelength of 590 nm by SPECTRAMAX GEMINIEM Microplate Reader (Molecular Devices, San Jose, CA, U.S.A.). Data were analyzed by SoftMax ProSoftware (Molecular Devices). In Figure 4B, the *Y*-axis is absorbance, where the scale was determined by the positive and negative controls. The ROS assay was repeated at least three times

### Morphology

Hepatocytes were cultured for 46 h with or without 100 mM of ethanol, and with 100 mM of ethanol and ADORA1-siRNA. Images were taken directly on hepatocyte-cultured plates using Leica DMi1 (phase-contrast microscope) and MC120 HD (camera), and analyzed using Leica Application Suite.

### Statistical analysis

All data are presented as the mean ± SEM. Statistical significance was determined by *t-*test or ANOVA.

## Results

### Chronological changes in gene expression at both mRNA and protein levels

**Figure 1:**
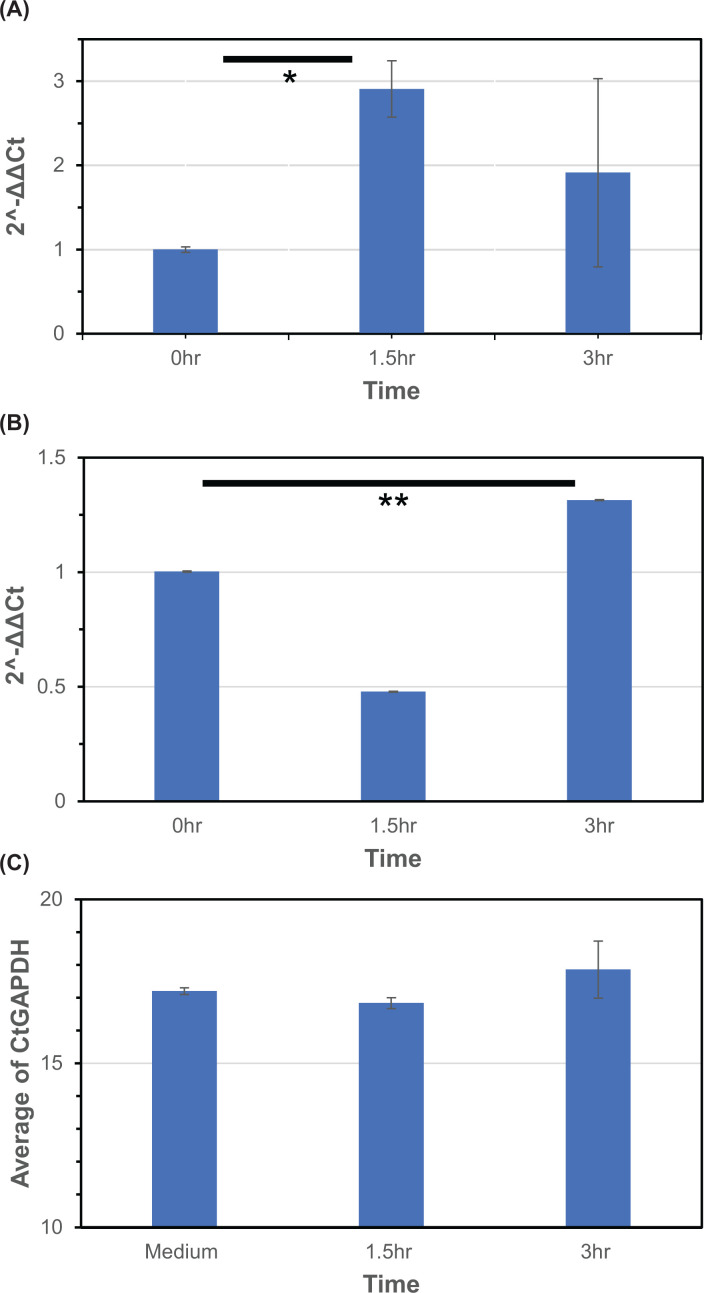
Chronological changes of ADORA1, caspase3 full-length, and GAPDH using quantitative real-time PCR (qPCR) Hepatocytes were cultured for 0 (control), 1.5, 3 or 6 h after the addition of ethanol at 100 mM. (**A**) ADORA1 gene expression. *X*-axis shows the time after adding ethanol into the culture medium at 100 mM. Values represent the mean and the standard error of mean (SEM); *T*-test, *P=0.0048. (**B**) Gene expression of caspase 3 full-length. *X*-axis shows the time after adding ethanol into the culture medium at 100 mM. Values represent the mean and the standard error of mean (SEM); *T*-Test, **P=0.0047. (**C**) Gene expression of GAPDH as a housekeeping gene. *X*-axis shows the time after adding ethanol into the culture medium at 100 mM. Values represent the mean and the standard error of mean (SEM). No significance was seen among three time points.

**Figure 2:**
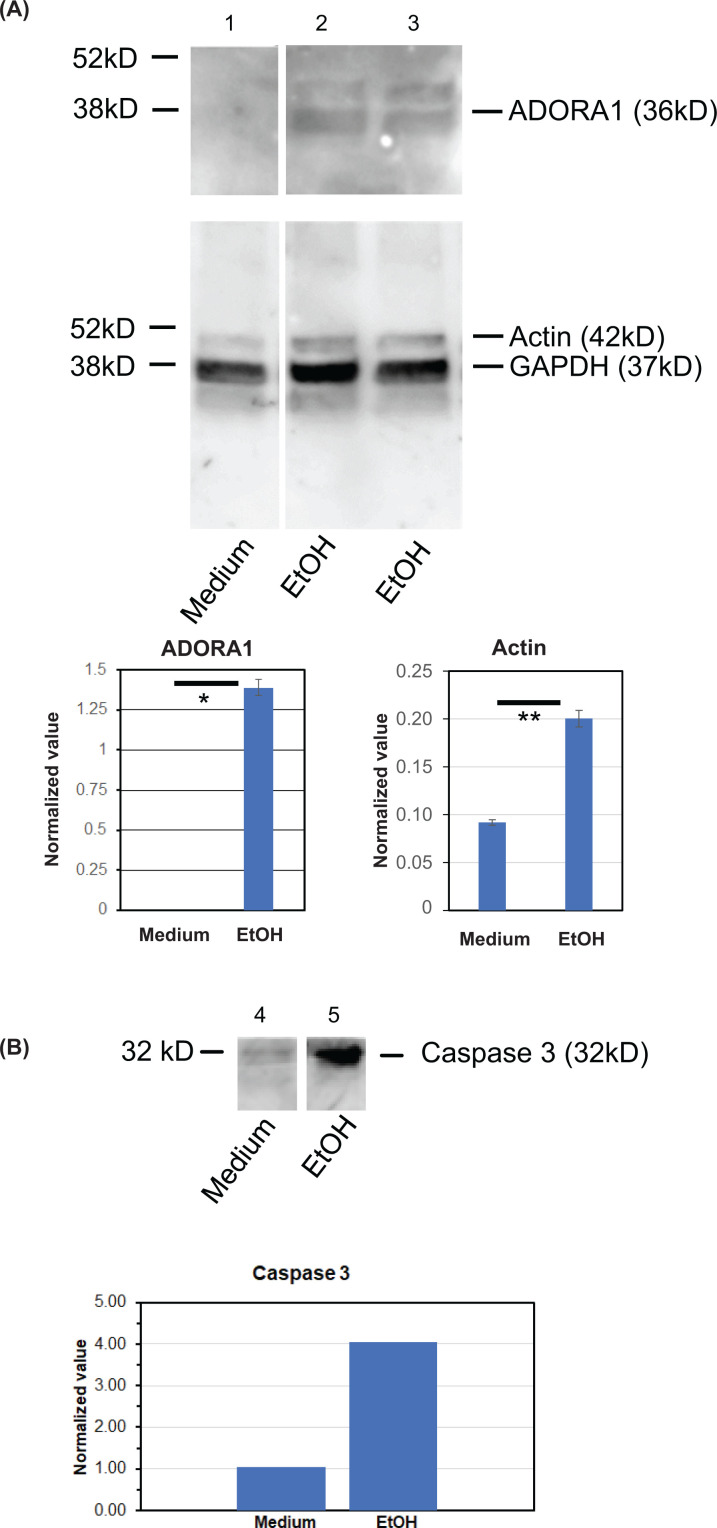
Western blot analysis of ADORA1, actin, GAPDH, and caspase 3 was performed on hepatocytes cultured for 48 h with and without 100 mM ethanol (EtOH) Each sample was normalized to GAPDH. Alternatively, normalization with total loaded amount of protein was also performed (Supplementary Figure S2). Poly-HRP conjugated secondary antibodies were used to enhance signals [8]. Lane 1: Cells cultured in medium only. Lane 2: Cells cultured with 100 mM ethanol. Lane 3: Cells cultured with 100 mM ethanol. Lane 4: Cells cultured in medium only. Lane 5: Cells cultured with 100 mM ethanol. (**A**) The expressions of ADORA1 and actin were measured in medium with and without 100 mM ethanol. No signals were detected for ADORA1 in medium only. The images are shown above and their quantified data are shown below. *T*-test analysis demonstrated that the both expressions of ADORA1 and actin were notably increased in the presence of ethanol (*P=0.04; **P=0.02). (**B**) The expression levels of caspase 3 were also measured in medium with and without 100 mM ethanol. The images are shown above, and the quantified data are shown below. The expression levels of caspase 3 were significantly increased in the presence of ethanol.

The kinetics of signal transduction cascades induced by ethanol were examined using quantitative real time PCR and Western blotting. Figure 1 shows quantitative real-time-PCR (qPCR) results showing chronological, ethanol induced changes at the mRNA level of ADORA1 (Figure 1A) and caspase 3 (Figure 1B) in iPSC derived hepatocytes which were cultured up to 6 h, along with ethanol stimulation at 100 mM. Figure 1A shows that ADORA1 mRNA levels peaked at 1.5 h after administering ethanol, trending down from 1.5 to 6 h. Meanwhile, caspase 3 peaked at 3 h (Figure 1B). For the protein expression levels, normalization was performed with GAPDH (Figure 2). Across the analyzed samples, GAPDH expression had been consistently high and stable, and much stronger than the other measured molecules. As a result, the observed minor variations in GAPDH levels across the time course should not affect GAPDH to serve as the housekeeping gene and the internal control. Furthermore, normalization with total loaded protein was also performed, ensuring that obtained normalized values are reliable (Supplementary Figure S2). In Figure 2A, protein expressions not only of ADORA1 but also of actin were notably up-regulated. In Figure 2B, caspase 3 protein expression was up-regulated as well.

### Signal transduction mechanisms and kinetics

**Figure 3:**
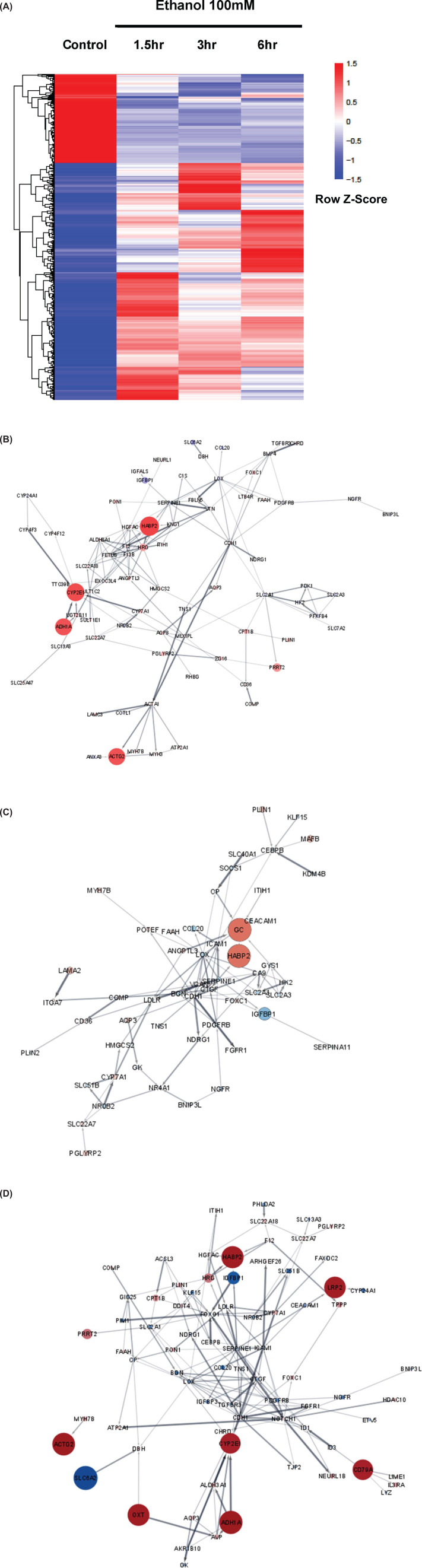
The analysis of signal transduction mechanisms and kinetics with NGS Samples were taken at 0 (control), 1.5, 3, and 6 h in the time course of cultures in the medium with 100 mM of ethanol. NovoSmart (Novogene), Cytocscape, UniProt, and KEGG PATHWAY Database were employed for the analysis. In (B) through (D), functional enrichments in the network of protein-coding genes through the time course are shown. (**A**) Heatmap comparison of gene expression at all four time points. A list of expressed gene symbols and names is available in Supplementary Figure S2. (**B**) Gene interactions and comparisons between 0 and 1.5 h. The width of each line is proportional to the strength of interaction between each neighbor. Circle sizes express the difference in expression intensity, where the size of the circles is proportional to the ratio (log2Fold) of the expression level at 1.5 h to that at 0 h. The color and intensity of each circle reflects the sign and magnitude of the values (log2Fold), where blue means it is negative and red is positive. -7.68 < log2Fold < 7.88. (**C**) Gene interactions and comparisons between 0 and 3 h. Circles express the difference in expression intensity, where the size of the circles reflects the ratio (log2Fold) of the expression level at 3 h to that at 0 h. The color and intensity of each circle reflects the sign and magnitude of the values (log2Fold), where blue means it is negative and red is positive; -7.05 < log2Fold < 7.79. (**D**) Gene interactions and comparisons between 0 and 6 h. Circles express the difference in expression intensity, where the size of the circles reflects the ratio (log2Fold) of the expression level at 6 h to that at 0 h. The color and intensity of each circle reflects the sign and magnitude of the values (log2Fold), where blue means it is negative and red is positive; -3.58 < log2Fold < 7.74.

Samples were taken at 0 (control), 1.5, 3, and 6 h in the time course of culture with 100 mM of ethanol, then subject to NGS. Figure 3A is the heatmap comparing each time point. Supplementary Table S1 is a list of expressed gene symbols and names in the same order that they appear on the heatmap. The map shows that the inactivated area in the control got activated and the activated area in control became silent after adding ethanol, and there are clusters of molecules particularly activated at each time point. Figure 3B–D are diagrams of gene-expressed proteins at 1.5, 3, and 6 h. Table 1 summarizes major genes/proteins expressed in response to ethanol stimulation. Most gene expression appeared at specific time points. However, some proteins were expressed at all three time points:

- CYP2E1 (cytochrome P450 monooxygenase) and ADH1 (alcohol dehydrogenase) were notably activated.
- HABP2 (Hyaluronan-binding protein 2), which is a coagulation factor VII-activating serine protease, was very highly activated [9–11].
- ACTA1 (Actin, α1) showed strong up-regulation at all three time points, although it is accompanied by two other molecules that appeared at only one of the three time points. CDC42BPG (CDC42-binding protein kinase γ) was mildly up-regulated only at 1.5 h (Figure 3B), and ARHGEF26 (Rho guanine nucleotide exchange factor 26) only at 6 h (Figure 3D).
- NOXA1 (A superoxide- producing NADPH oxidase), which induces the production of reactive oxygen species (ROS), was also mildly up-regulated.

Moreover, some molecules were activated mainly at 6 h (Figure 3D):

- ID1 (DNA-binding protein inhibitor ID-1) was very highly activated. ID1 positively regulates actin filament bundle assembly and apoptosis [12].
- DDIT4 (DNA damage-inducible transcript 4 protein) is significantly up-regulated. DDIT4 induces p53/TP53-mediated apoptosis by inhibiting the activity of the ‘mammalian target of rapamycin complex 1’ (mTORC1), and is activated in response to cellular stress including DNA damage [13].
- OXT (oxytocin-neurophysin 1) was strongly up-regulated. OXT is a prepropeptide which is cleaved into the two chains: oxytocin and neurophysin I. High concentrations of oxytocin inhibit the growth of liver [14]. Oxytocin also up-regulates prostaglandin secretion.

Furthermore, PTGDR2 (Prostaglandin D2 receptor 2) was mildly activated at 1.5 and 6 h (Figure 3B,D). This receptor’s activation is involved in inflammation responses. Also, KNG1 (Kininogen-1) is mildly up-regulated at 1.5 h (Figure 3B). It plays an important role in blood coagulation and functions as a mediator of inflammatory response including release of prostaglandins.

Meanwhile, quite a few molecules remained suppressed after ethanol administration (Figure 3):

- SLC2A1 (solute carrier family 2, facilitated glucose transporter member 1) was remarkably repressed, causing glucose uptake to be signally reduced;
- LYZ (Lysozyme C) was remarkably repressed. More than 10% of cellular glycogen is located within the lysosome, which becomes a final energy source in stress situations including starvation with the rupture of lysosomes [15]. LYZ repression is proposed to accelerate the aggravation of starvation of hepatocytes, as this final energy store becomes much less available despite in the starvation state.
- IGFBP1 (insulin-like growth factor binding protein 1) was strikingly repressed, leading to down-regulation of glucose uptake and unequivocal facilitation of programmed cell death, as IGF-1 is one of the most potent activators of the AKT signaling pathway [16].
- SERPINE1 – serpin peptidase inhibitor, clade E (nexin, plasminogen activator inhibitor type 1), member 1 – was strongly down-regulated. In the presence of vitronectin (VTN), SERPINE1 was found to be an effective thrombin inhibitor [17]. In the present study, VTN was significantly up-regulated at 1.5 h, possibly due to positive feedback brought about by the down-regulation of SERPINE1. SERPINE1 interacts with IGFBP1 signaling from SERPINE1 to IGFBP1. It seems that the down-regulation of SERPINE1 intensifies the down-regulation of IGFBP1.

Moreover, CEBPB (CCAAT/enhancer binding protein [C/EBP], beta) showed very strong repression at 3 and 6 h (Figure 3C,D), especially at 6 h. This molecule plays a significant role in the gluconeogenic pathway and in the regulation of acute-phase reaction and inflammation. Therefore, CEBPB repression should significantly reduce gluconeogenesis, leaving hepatocytes with much less available glucose and more aggressive inflammation.

PHLDA2 (Pleckstrin homology-like domain family A member 2) was moderately down-regulated at 3 and 6 h (Figure 3C,D). It controls glycogen storage [18].

Here, PHLDA2 down-regulation together with LYZ, play an important role in glycogen storage and suppression of glycogenolysis, worsening the starvation state.

SOAT2 (sterol O-acyltransferase 2: cholesterol acyltransferase 2) was markedly down-regulated at 3 h (Figure 3C). It produces intracellular cholesterol esters for lipoprotein secretion from hepatocytes [19]. Therefore, its down-regulation seems to induce the retention of lipids inside the hepatocytes. Moreover, GK (ATP:glycerol 3-phosphotransferase) was significantly suppressed at 3 and 6 h (Figure 3C,D). This is a key enzyme in the regulation of glycerol uptake.

IER3 (radiation-inducible immediate-early gene IEX-1) was moderately suppressed at 3 h (Figure 3C). ERK1/2 is activated by IER3 [20–22]. CTGF (synonym of CCN2, CCN family member 2) was significantly suppressed at 3 and 6 h (Figure 3C,D). This molecule positively regulates the cascades of ERK1 and ERK2 as well as JNK.

### Use of actin as a damage marker and the role of ADORA1 in alcohol-intoxicated hepatocytes using ADORA1-siRNA

**Figure 4:**
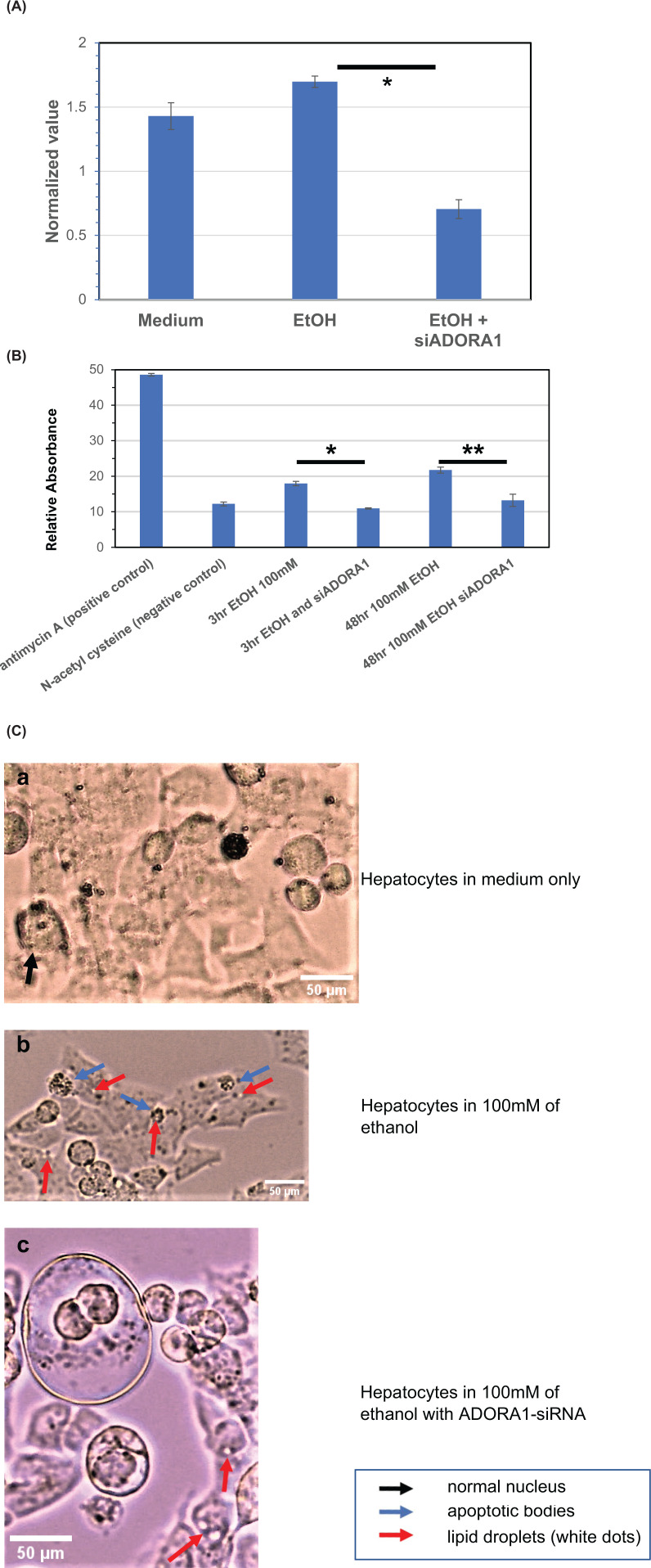
Evaluation of ADORA1-siRNA transfection into hepatocytes Hepatocytes were transfected with ADORA1-siRNA followed by culture in medium containing 100 mM of ethanol. This figure presents the results of Western blot analysis (**A**), reactive oxygen species (ROS) assay (**B**) and morphological evaluation (**C**). (A) Hepatocytes transfected with ADORA1-siRNA were then cultured for 48 h in 100 mM ethanol (EtOH) along with the cells cultured in medium only and the cells cultured only in 100 mM ethanol (EtOH). Each sample was normalized to GAPDH of the same group. Poly-HRP conjugated secondary antibodies were used to enhance signals. Actin expression cultured with 100 mM ethanol notably decreased with the transfection of ADORA1-siRNA into the hepatocytes. *T*-test: *P=0.007. (**B**) Reactive oxygen species (ROS) assay with ADORA1-siRNA. The hepatocytes were cultured for 3 or 48 h at 100 mM of ethanol with or without the transfection of ADORA1-siRNA into the hepatocytes. Antimycin A was used as the positive control and N-acetyl cysteine as the negative control. The fluorescence was measured using exciting wavelength of 485 nm and emission wavelength of 590 nm by SPECTRAMAX GEMINIEM (Molecular Devices). Data were analyzed with SoftMax ProSoftware (Molecular Devices). *Y*-axis shows absorbances where the scale was determined by the positive and negative controls. The production of ROS was significantly increased at 48 h compared with 3 h in the hepatocytes cultured with 100 mM ethanol (*P=0.02). The production of ROS was significantly decreased in the hepatocytes cultured with 100 mM ethanol and the transfection of ADORA1-siRNA at both 3 and 48 h (**P=0.04). (**C**) Phase-contrast microscopy was used to visualize the morphology of hepatocytes cultured for 46 h with medium only, 100 mM ethanol, or 100 mM ethanol and ADORA1-siRNA. Apoptotic bodies and lipid droplets were observed in the hepatocytes cultured with 100 mM ethanol (b). The number of apoptotic bodies and lipid droplets was significantly decreased in the hepatocytes cultured with 100 mM ethanol and ADORA1-siRNA (c). No such abnormalities were observed in the hepatocytes cultured with medium only (a).

Actin is known as one of the most abundant proteins in hepatocytes. Therefore, changes in actin expression levels were anticipated to be more apparent than those of other proteins. In Figure 4A, hepatocytes transfected with ADORA1-siRNA exhibited a significant down-regulation of actin protein expression after 48 h of culture in 100 mM ethanol, compared with cells cultured only in 100 mM ethanol for the same duration. Figure 4B shows the results of a ROS assay under the same conditions as Figure 4A. ROS production was significantly higher at 48 h than at 3 h of incubation with 100 mM of ethanol. However, the amount of ROS was significantly lower in the ADORA1-siRNA transfected hepatocytes at both 3 and 48 h. For morphologic analysis of the hepatocytes, images were taken using a phase-contrast light microscope (Figure 4C). Apoptotic bodies and lipid droplets were visible in the cells that were cultured in ethanol for 46 h (Figure 4C,b). These abnormalities were less discernible in the cells that were transfected with ADORA1-siRNA (Figure 4C,c). No such abnormalities were seen in the cells that were cultured in medium only (Figure 4C,a). These findings suggest that blocking ADORA1 with siRNA can reduce the production of ROS and apoptosis in the hepatocytes cultured with ethanol; and that actin can be used as a biomarker of damage in alcohol-intoxicated hepatocytes.

## Discussion

The findings of the present study demonstrated that massive ethanol influx into hepatocytes causes a variety of complex reactions. CYP2E1 is a membrane protein expressed in hepatocytes acting as the gateway for ethanol to enter the cascade. It has been reported that transcriptional induction of CYP2E1 occurs with high levels of ethanol [23]. Interestingly, another protein, NOXA1 was readily activated along with CYP2E1 in response to ethanol stimulation, and remained activated at all three time points. This correlates well with the rapid elevation of ROS production brought about by the surge of a toxically high concentration of ethanol (Figure 4C).

CDC42BPG is a serine/threonine-protein kinase, and ARHGEF26 activates Rho GTPase by promoting the exchange of GDP for GTP. Cdc42 is a GTPase of the Rho family involved in various signaling pathways which controls diverse cellular functions, including actin assembly and rearrangement. It is known that actin is up-regulated in response to stresses and form stress fibers [24]. Therefore, CDC42BPG and ARHGEF26 acting together are postulated to activate actin formation. ID1 is assumed to boost actin accumulation.

DDIT4 is activated in response to many stress stimuli including DNA damage. Therefore, it is proposed that DDIT4 up-regulation was induced at the later time point through apoptosis accompanying DNA damage, and through stresses from starvation and inflammation by the rapid influx of the high concentration of ethanol. In turn, ID1 up-regulation induces apoptosis through an intrinsic (mitochondria-mediated) pathway (KEGG apoptosis pathway: https://www.genome.jp/pathway/hsa04210). Thus, once the cycle of this sequence is established, apoptosis is assumed to rapidly accelerate, predisposing the hepatocytes to fulminant and fatal hepatitis. Hence, a number of stresses triggered by the ethanol surge are likely to have induced DDIT4 and ID1 up-regulation observed at the later time point.

In this study, an acute and high rise in the concentration of ethanol forced hepatocytes to suffer from increasingly low levels of glucose, brought about by the various described molecules related to this ethanol surge. This surge is postulated to starve hepatocytes thus effectively and hence further aggravate inflammation and apoptosis. Also, both IER3 and CTGF suppression are postulated to synergistically facilitate apoptosis by the downstream suppression of MAP kinase pathways.

In the meantime, it is known that acute hepatitis accompanies steatosis [25]. Marked suppression of two enzymes, SOAT2 and GK, is assumed to play an important role in increasing lipid levels in the affected liver, bringing about hepatic steatosis.

HABP2 and SERPINE1 appear to synergistically facilitate the formation of blood clots, and these two proteins are further boosted by KNG1. It has been reported that rapid and large ingestion of ethanol predisposes us to grave thrombosis and embolism in vessels, especially in the coronary arteries [26].

The effects of suppressing these three enzymes might be part of a mechanism explaining these clinical phenomena.

The results from qPCR and Western blotting indicate that ADORA1 is located further upstream than caspase 3 on the stream of signal transduction that starts with ethanol intake into the cells. The actin amount seems to correlate well with the severity of stresses. Therefore, actin is suggested as an appropriate biomarker, since it is abundant and changes are believed to have reliable sensitivity and specificity to reflect stress severity.

**Figure 5:**
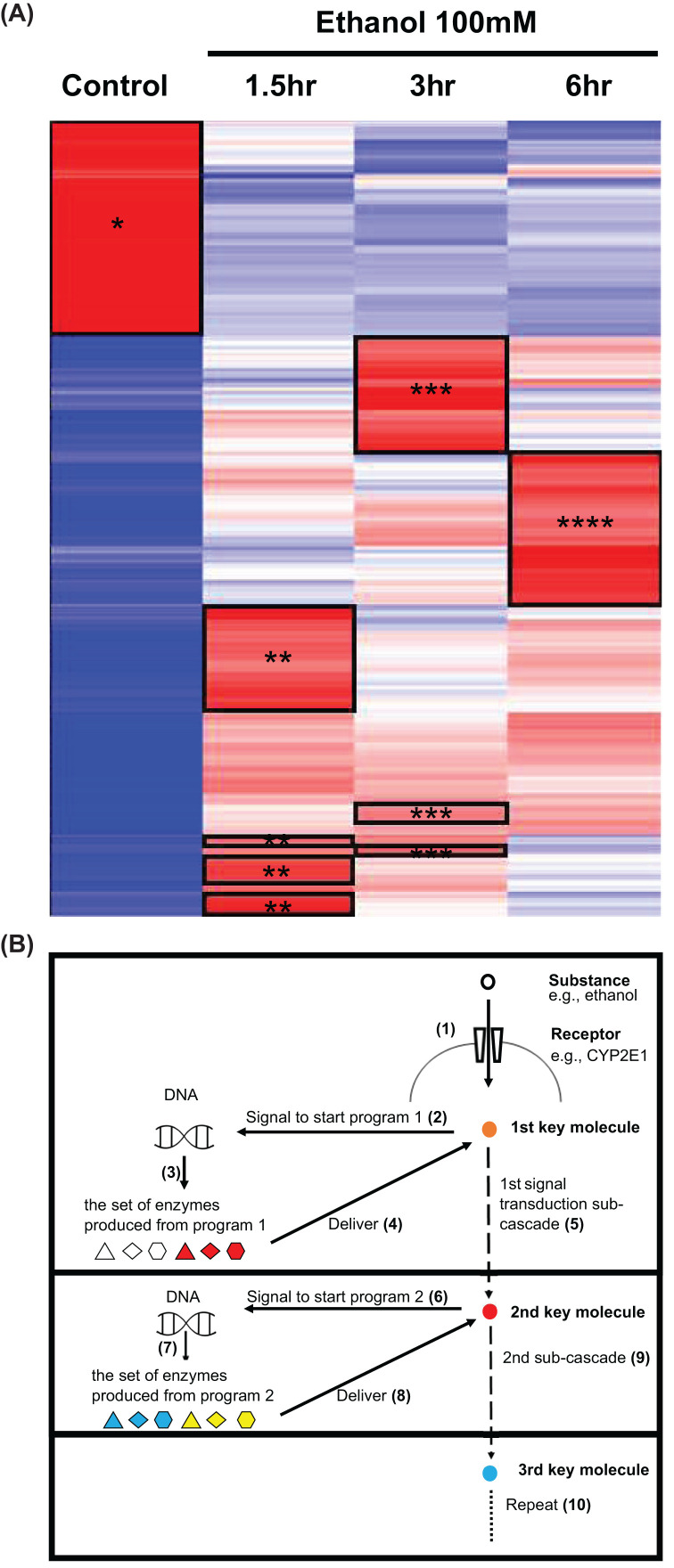
A hypothesis for the system of ethanol induced signal transduction This figure shows a hypothesis for the system of ethanol-induced signal transduction. The hypothesis is based on the results of NGS data analysis (**A**) and a diagram (**B**). (A) Heatmap obtained from analysis of NGS data on gene expression in hepatocytes cultured in medium with 100 mM of ethanol (identical to Figure 3A): The areas framed in black are up-regulated clusters associated with specific time points. One asterisk indicates the control group where the hepatocytes were cultured only in medium. Two asterisks indicate the group where the hepatocytes were cultured in 100 mM of ethanol for 1.5 h. Three asterisks represent the group where the hepatocytes were cultured in 100 mM of ethanol for 3 h. Four asterisks represent the group where the hepatocytes were cultured in 100 mM of ethanol for 6 h. (**B**) The diagram illustrates the hypothesis: (1) Substrate (ethanol in this case) enters the cell through its receptors (CYP2E1 in this case) and undergoes first reactions in the pathway. (2) A first key molecule is produced and sends a signal to run the first program in the nuclei. (3) The initial set of enzymes is produced by the program. (4) This first set of enzymes is delivered to the first key molecule. (5) The reactions of the first sub-cascade are executed. (6) A second key molecule is produced and sends a signal to run a second program in the nuclei. (7) A second set of enzymes is produced by the program. (8) The second set of enzymes is delivered to the second key molecule. (9) The reactions in a second sub-cascade are executed. (10) A third key molecule is produced and analogous cycles are repeated until the end products are produced.

On the heatmap (Figure 5A), each time point has its own cluster(s) of up-regulated gene expression contrasting with other time points, so there is no uniform pattern of gene expression during the time course examined. It seems that when pathways are activated, each sub-cascade is sequentially activated. Based on this observation, it is hypothesized that overall cascade is sectioned (Figure 5B) into a series of sub-cascades. In this hypothesis, after a substance enters a cell, a first key molecule is produced that signals for the nuclei to run a pre-determined program which activates genes governing the first section of the pathway. A first set of enzymes thus made reaches the first key molecule, leading to rapid signal transduction in the first sub-cascade. This first sub-cascade produces a second key molecule. Then, this second key molecule similarly activates the DNAs to produce the prerequisite enzymes that get delivered to implement a second sub-cascade. This yields a third key molecule. After executing each sub-cascade, final products are thought to cause observable outcomes or symptoms such as inflammation, fever, or clot formation.

Arguably, the gene expression pattern (Figure 5A) is separated into two groups. Genes up-regulated in the medium only are down-regulated under ethanol stimulation, whereas genes up-regulated under ethanol stimulation are down-regulated in the medium only. This means proteins that reacted to ethanol are primarily induced without conserved proteins. This may explain why there are time lags following ethanol ingestion before the expression of symptoms in patients suffering after binge drinking.

ADORA1-siRNA showed significant suppression of actin protein expression and ROS suppression that clearly follows ADORA1-siRNA transfection at both 3 and 48 h. It has been reported that the apoptosis pathway consists of two distinct signal transduction streams, and that one is activated through caspases and the other through ROS generation (KEGG apoptosis pathway, URL: https://www.genome.jp/pathway/hsa04210). These data indicate that adenosine A1 receptors in the liver contribute to apoptosis leading to liver damage in acute alcoholism through both intrinsic pathway and reactive oxygen species activity. Animal studies should be an appropriate and practical next step to examine the magnitude of ADORA1-siRNA effects that can be elicited clinically.

In the present study, physiological hepatocytes are shown to ultimately self-destruct in apoptosis, and the stresses appear to synergistically build up and accelerate the gravity of liver damage, following well-orchestrated and synergistic signal transduction pathways set in motion by an acute large bolus influx of ethanol. It is assumed that the molecules brought about by ethanol stimulation are induced proteins and not conserved proteins. It is hypothesized that each sub-cascade is executed promptly with delivery of the set of prerequisite enzymes, and these sub-cascade processes take place in sequence.

In conclusion, the results of these experiments suggest that ADORA1 plays a significant role in ethanol-induced metabolism in hepatocytes. ADORA1 is involved in the regulation of ROS production and apoptosis. Blocking ADORA1 with siRNA can reduce the production of ROS and apoptosis in the hepatocytes cultured with ethanol. Actin is proposed to be an appropriate biomarker for evaluation of the degree of liver damage.

## Supplementary Material

Supplementary Figures S1-S2

Supplementary Table S1

## Data Availability

The datasets generated and analyzed during the current study are available in the NCBI SRA repository with accession number SRX19524277.

## Abbreviations

ANOVA: analysis of variance
ARHGEF26: Rho guanine nucleotide exchange factor (GEF) 26
CDC42BPG: CDC42-binding protein kinase γ
CEBPB: CCAAT/enhancer binding protein β
CTGF: connective tissue growth factor
CYP2E1: cytochrome P450 family 2 subfamily E member 1
DDIT4: DNA-damage-inducible transcript 4
GAPDH: glyceraldehyde 3-phosphate dehydrogenase
GK: glycerol kinase
HABP2: hyaluronan-binding protein 2
HLA: human leukocyte antigen
HRP: horseradish peroxidase
ID1: inhibitor of DNA binding 1
IER3: immediate early response 3
IGFBP1: insulin-like growth factor binding protein 1
KNG1: kininogen-1
LYZ: lysozyme
NOXA1: NADPH oxidase activator 1
OXT: oxytocin/neurophysin I prepropeptide
PHLDA2: pleckstrin homology-like domain family A member 2
PTGDR2: prostaglandin D2 receptor 2
SERPINE1: plasminogen activator inhibitor-1
SLC2A1: solute carrier family 2, facilitated glucose transporter member 1
SOAT2: sterol O-acyltransferase 2
