# Supplementary material for: Elucidating the role of intrinsic adenosine A1 receptors in acute alcoholism using human-induced pluripotent stem cell-derived hepatocytes
Source: Biosci Rep. 2024 Mar 21;44(3):BSR20231682. doi: 10.1042/BSR20231682 (PMC10958140; doi:10.1042/BSR20231682)
Supplement: Supplementary Figures S1-S2 [file BSR-2023-1682_supp.pdf]

Supplementary Material

Elucidating the role of intrinsic adenosine A1 receptors in acute alcoholism using human induced pluripotent stem cell-derived hepatocytes

Takako Nagata, Yuning George Huang

List of material included:

Figure S1: Role of adenosine A1 receptors in exacerbation of inflammation and liver damage under excessive alcohol consumption

Figure S2: Western blot analysis of ADORA1 and actin (supplement data for Figure 2A)

Table S1: List of expressed gene symbols and names in the order they appear on the heatmap in Figure 3A

Figure S1

A

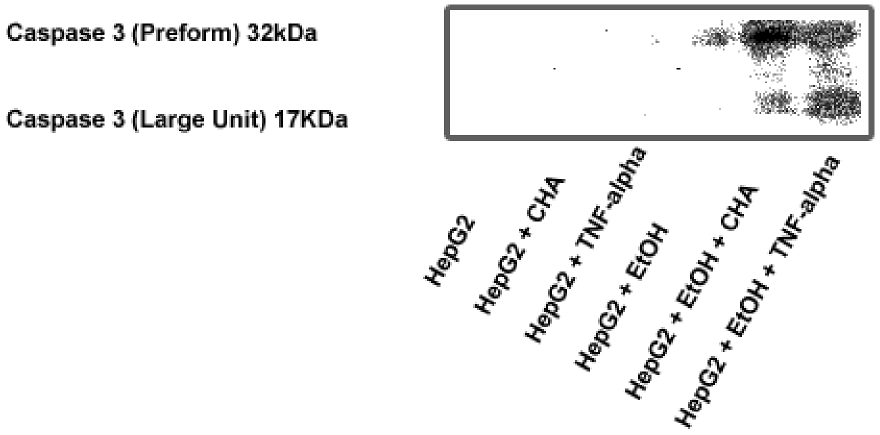

B

TNF-α (pg/ml)	
Wildtype	92.5 ± 10.6
A1R KO	11.9 ± 5.9

Figure S1: Role of adenosine A1 receptors in exacerbation of inflammation and liver damage under excessive alcohol consumption. In vivo, adenosine A1 receptor knockout mice (A1R KO) with C57BL/6 background (8wk old, male, 25g), and wild-type from the same colonies were employed. N6-cyclohexyladenosine (CHA), A1 agonist, from Tocris, was intraperitoneally injected at 0.3 mg/kg. At 30 min, ethanol (EtOH) was intraperitoneally injected at 3.6 g/kg. Sera at 1.5 hours were assayed for TNF-α as an inflammation level indicator. In vitro, HepG2 (liver hepatocellular carcinoma) cell line at 3 x 10^5 cells / ml / well, was cultured with ethanol at 100mM or with EtOH at 100mM and CHA at 10^{-5}M. TNF-α (1ng/ml) was used to make the positive controls. The cells were harvested after 45-hour culture to assess TNF-α and caspase 3 (preform and the large unit of activated form) using western blotting. Caspase 3 was detected with anti-caspase 3 antibody from Santa-Cruz Biotechnology. Total protein was measured with Protein Assay from Bio-Rad. 10ug of protein was loaded in each lane.

(A) Caspase 3 expression in HepG2 with EtOH and CHA: Co-stimulation of EtOH and CHA caused significantly higher expression of preform caspase 3 which was even higher than the positive control with EtOH and TNF-α, while EtOH stimulation alone caused mild increase in preform caspase 3. Activated caspase 3 was also induced by co-stimulation of EtOH and CHA, while no such induction was observed with EtOH alone. Total protein yielded from cultures with EtOH + CHA and with EtOH + TNF-α at 45 hr was below 65 % of that from HepG2 alone. TNF-α was not detected in the samples from HepG2 alone, HepG2 + CHA, HepG2 + EtOH, or HepG2 + EtOH + CHA).

(B) TNF-α with EtOH in C57BL/6 mice & A1R KO: At 1.5 hours after ethanol injection, TNF-α values were markedly higher in the wild type group than in the A1R KO group (n = 5 to 6 per group).

Figure S2

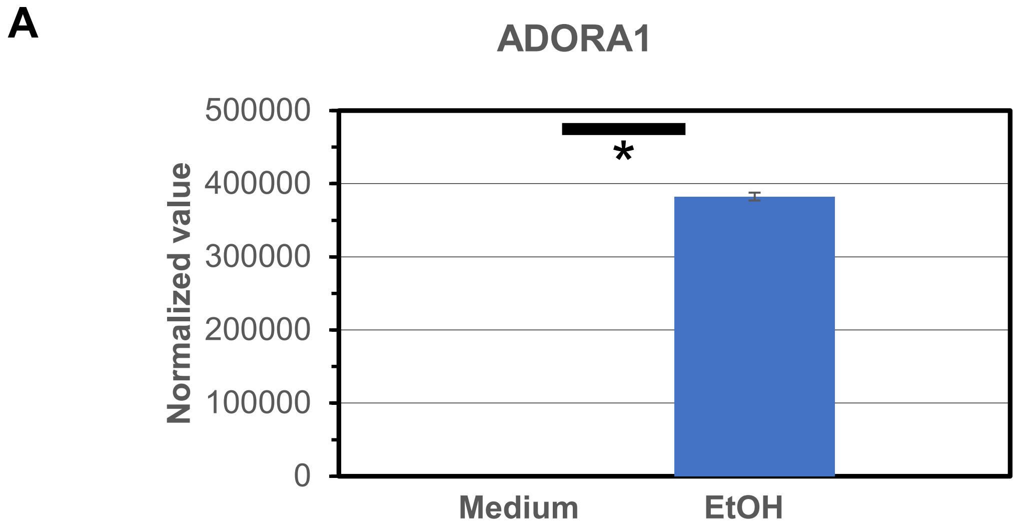

A
ADORA1
500000
400000
300000
200000
100000
0
Normalized value
*
Medium
EtOH

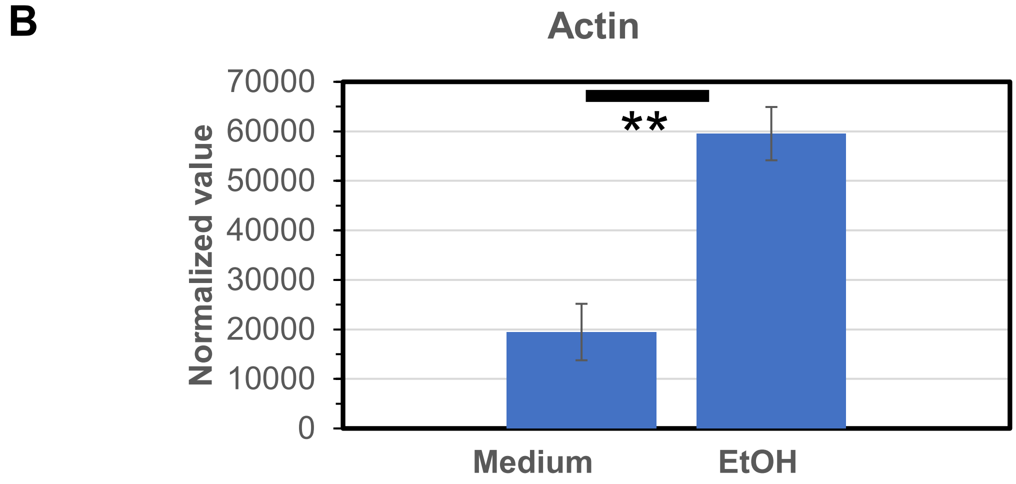

B
Actin
70000
60000
50000
40000
30000
20000
10000
0
Normalized value
**
Medium
EtOH

Figure S2: Western blot analysis of ADORA1 and actin (supplement data for Figure 2A). Each measured volume of signals for ADORA1 and actin was respectively normalized to the amount of total loaded protein. Both ADORA1 and actin demonstrated equivalent results to Figure 2A.

(A) ADORA1 protein expression was normalized to its total loaded amount (μg). T-test analysis showed that ADORA1 protein expression was remarkably increased in the presence of ethanol (*p= 0.004).

(B) Actin protein expression was normalized to its total loaded amount (μg). T-test analysis showed that actin protein expression was significantly increased in the presence of ethanol (**p= 0.04).

Table S1: List of expressed gene symbols and names in the order they appear on the heatmap in Figure 3A (Excel file).

Supplementary Material for western blotting

Elucidating the role of intrinsic adenosine A1 receptors in acute alcoholism using human induced pluripotent stem cell-derived hepatocytes

Takako Nagata, Yuning George Huang

List of material included:

Figure S2: Unprocessed original Images of western blots

Figure S2

A

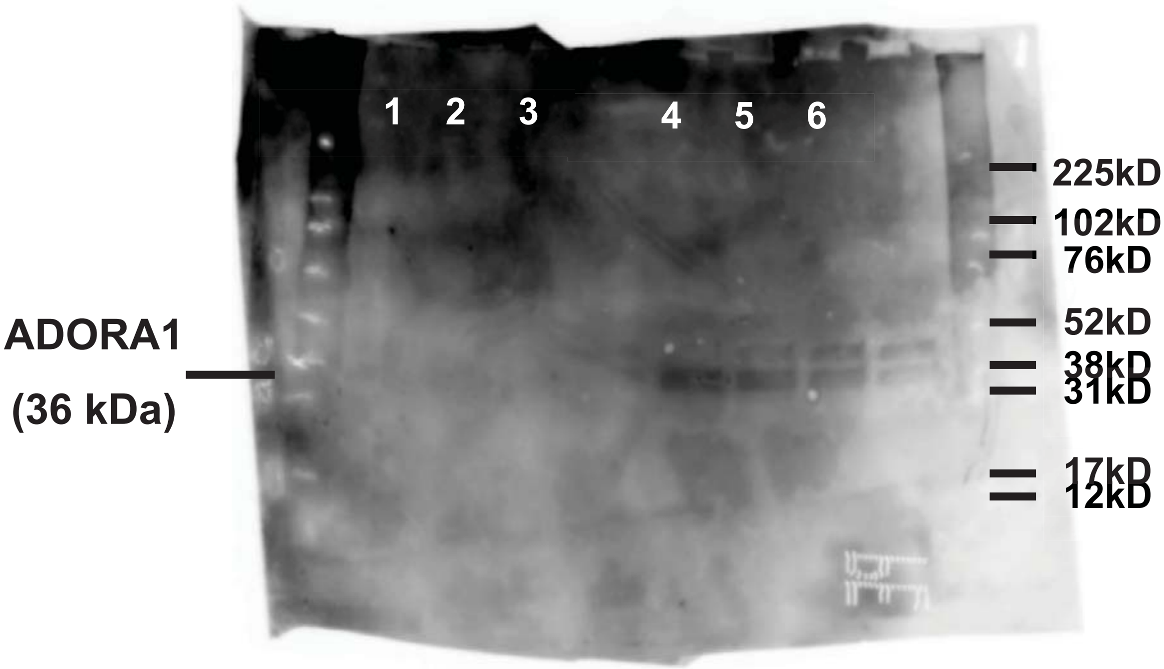
1
2
3
4
5
6
225kD
102kD
76kD
52kD
38kD
31kD
17kD
12kD
ADORA1
(36 kDa)

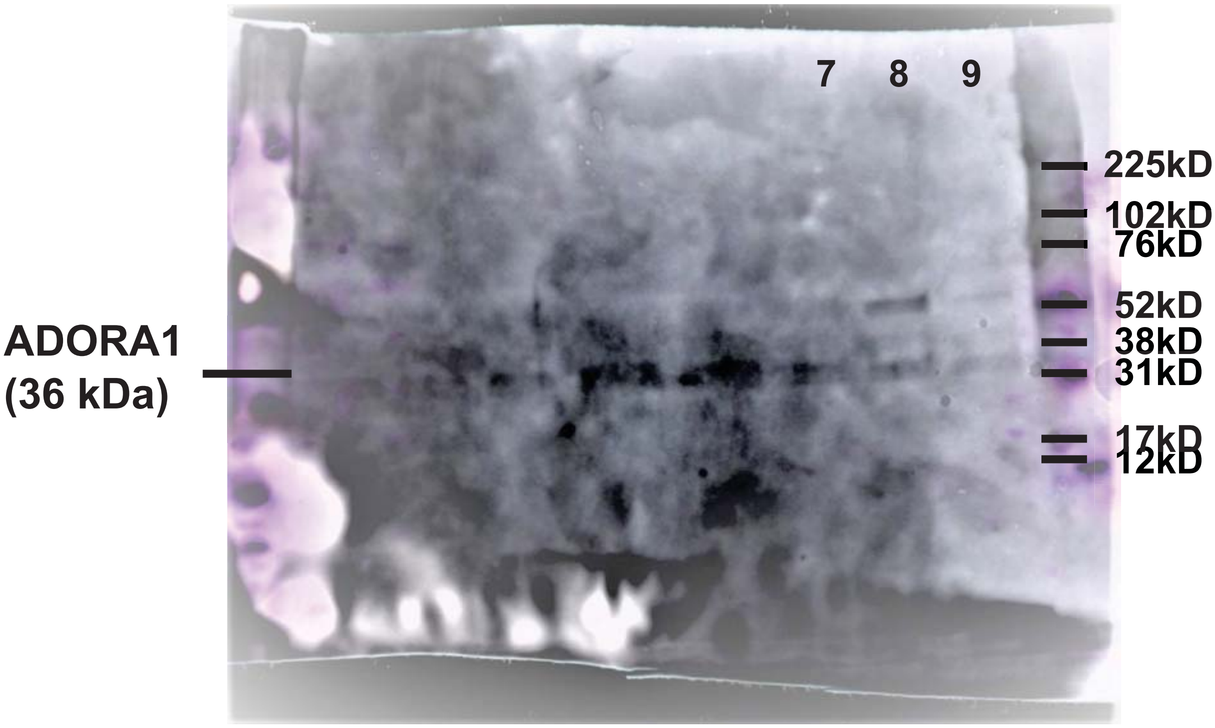
7
8
9
225kD
102kD
76kD
52kD
38kD
31kD
17kD
12kD
ADORA1
(36 kDa)

B

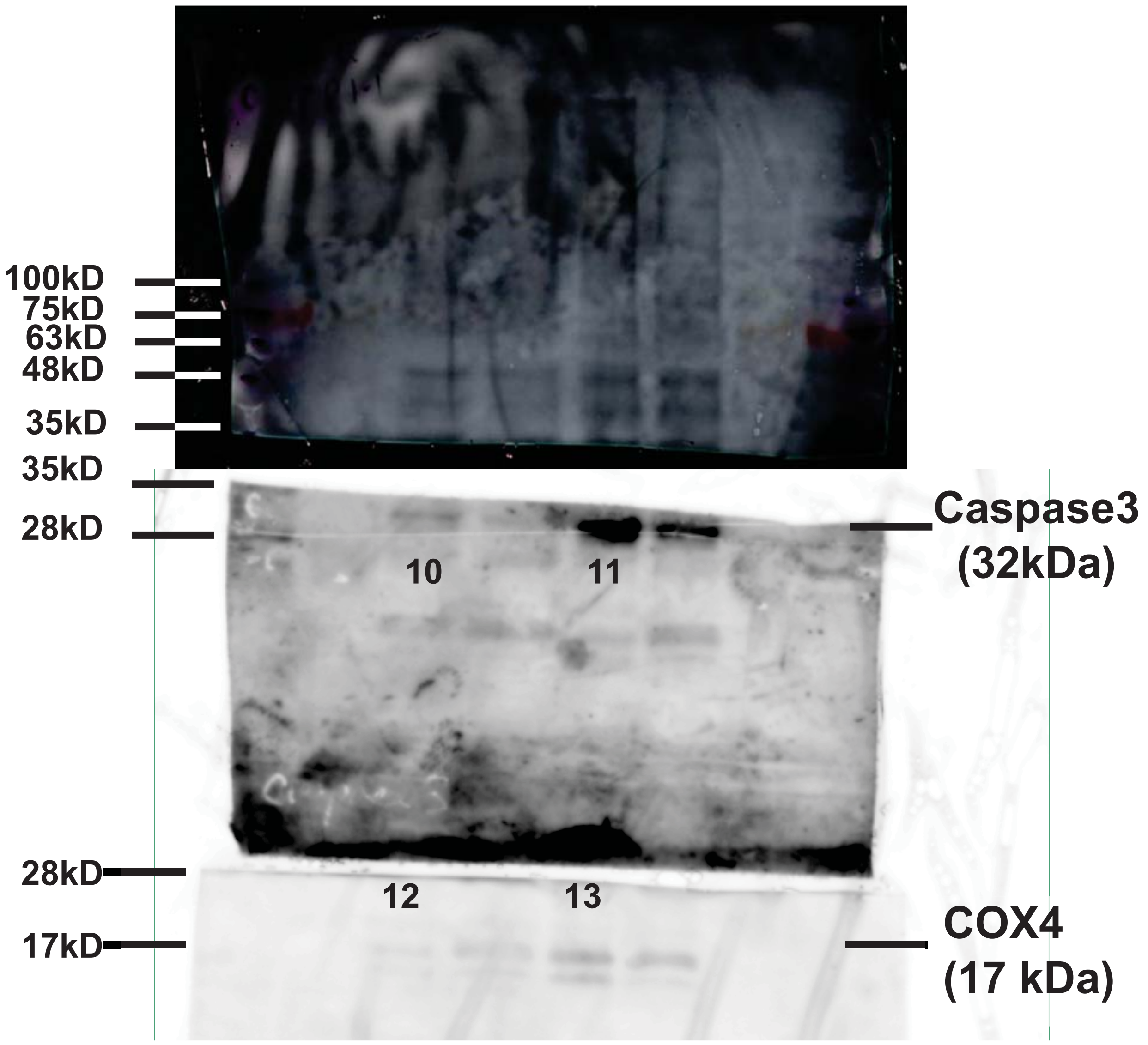

100kD
75kD
63kD
48kD
35kD
35kD
28kD
Caspase3
(32kDa)
10
11
28kD
12
13
17kD
COX4
(17 kDa)

C

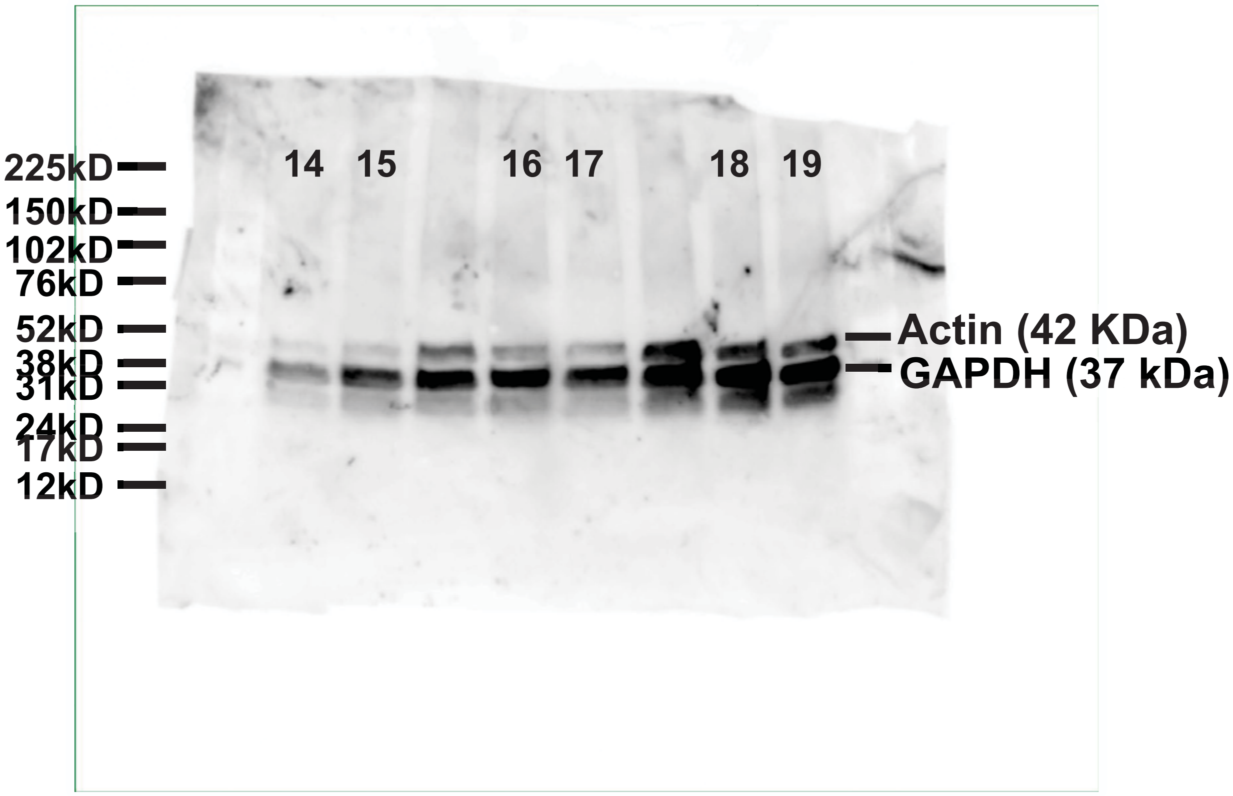
225kD
150kD
102kD
76kD
52kD
38kD
31kD
24kD
17kD
12kD
14
15
16
17
18
19
Actin (42 KDa)
GAPDH (37 kDa)

Figure S2: Original and unprocessed blots for Figure 2 and 4: (A) ADORA1 (1-3: cultured in medium only, 4-6: cultured in 100mM of ethanol, 7-9: cultured in 100mM of ethanol and siADORA1); (B) caspase 3 (10: cultured in medium only, 11: cultured in 100mM of ethanol); cox4 (12: cultured in medium only, 13: cultured in 100mM of ethanol); (C) actin and GAPDH (14-15: cultured in medium only, 16-17: cultured in 100mM of ethanol, 18-19: cultured in 100mM of ethanol and siADORA1).
